# Lesion covariance networks reveal proposed origins and pathways of diffuse gliomas

**DOI:** 10.1093/braincomms/fcab289

**Published:** 2021-12-04

**Authors:** Ayan S Mandal, Rafael Romero-Garcia, Jakob Seidlitz, Michael G Hart, Aaron F Alexander-Bloch, John Suckling

**Affiliations:** Department of Psychiatry, Brain Mapping Unit, University of Cambridge, Cambridge, CB2 0SZ, UK; Department of Psychiatry, Brain-Gene Development Lab, Perelman School of Medicine, University of Pennsylvania, Philadelphia, PA 19104, USA; Department of Psychiatry, Brain Mapping Unit, University of Cambridge, Cambridge, CB2 0SZ, UK; Department of Psychiatry, Brain-Gene Development Lab, Perelman School of Medicine, University of Pennsylvania, Philadelphia, PA 19104, USA; Department of Child and Adolescent Psychiatry and Behavioral Science, Children’s Hospital of Philadelphia, Philadelphia, PA 19104, USA; Department of Psychiatry, Brain Mapping Unit, University of Cambridge, Cambridge, CB2 0SZ, UK; Academic Division of Neurosurgery, Department of Clinical Neurosciences, University of Cambridge, Cambridge, CB2 0SZ, UK; Department of Psychiatry, Brain-Gene Development Lab, Perelman School of Medicine, University of Pennsylvania, Philadelphia, PA 19104, USA; Department of Child and Adolescent Psychiatry and Behavioral Science, Children’s Hospital of Philadelphia, Philadelphia, PA 19104, USA; Department of Psychiatry, Brain Mapping Unit, University of Cambridge, Cambridge, CB2 0SZ, UK

**Keywords:** glioma, structural connectivity, functional connectivity, subventricular zone, neural stem cells

## Abstract

Diffuse gliomas have been hypothesized to originate from neural stem cells in the subventricular zone and develop along previously healthy brain networks. Here, we evaluated these hypotheses by mapping independent sources of glioma localization and determining their relationships with neurogenic niches, genetic markers and large-scale connectivity networks. By applying independent component analysis to lesion data from 242 adult patients with high- and low-grade glioma, we identified three lesion covariance networks, which reflect clusters of frequent glioma localization. Replicability of the lesion covariance networks was assessed in an independent sample of 168 glioma patients. We related the lesion covariance networks to important clinical variables, including tumour grade and patient survival, as well as genomic information such as molecular genetic subtype and bulk transcriptomic profiles. Finally, we systematically cross-correlated the lesion covariance networks with structural and functional connectivity networks derived from neuroimaging data of over 4000 healthy UK BioBank participants to uncover intrinsic brain networks that may that underlie tumour development. The three lesion covariance networks overlapped with the anterior, posterior and inferior horns of the lateral ventricles respectively, extending into the frontal, parietal and temporal cortices. These locations were independently replicated. The first lesion covariance network, which overlapped with the anterior horn, was associated with low-grade, isocitrate dehydrogenase -mutated/1p19q-codeleted tumours, as well as a neural transcriptomic signature and improved overall survival. Each lesion covariance network significantly coincided with multiple structural and functional connectivity networks, with the first bearing an especially strong relationship with brain connectivity, consistent with its neural transcriptomic profile. Finally, we identified subcortical, periventricular structures with functional connectivity patterns to the cortex that significantly matched each lesion covariance network. In conclusion, we demonstrated replicable patterns of glioma localization with clinical relevance and spatial correspondence with large-scale functional and structural connectivity networks. These results are consistent with prior reports of glioma growth along white matter pathways, as well as evidence for the coordination of glioma stem cell proliferation by neuronal activity. Our findings describe how the locations of gliomas relate to their proposed subventricular origins, suggesting a model wherein periventricular brain connectivity guides tumour development.

## Teaser

Mandal et al. report that glioma localization patterns coincide with structural and functional connectivity networks disseminating from the SVZ. These findings support a model wherein periventricular brain connectivity guides glioma development from neurogenic niches into distributed cortical locations.

## Graphical Abstract

**Graphical Abstract:**
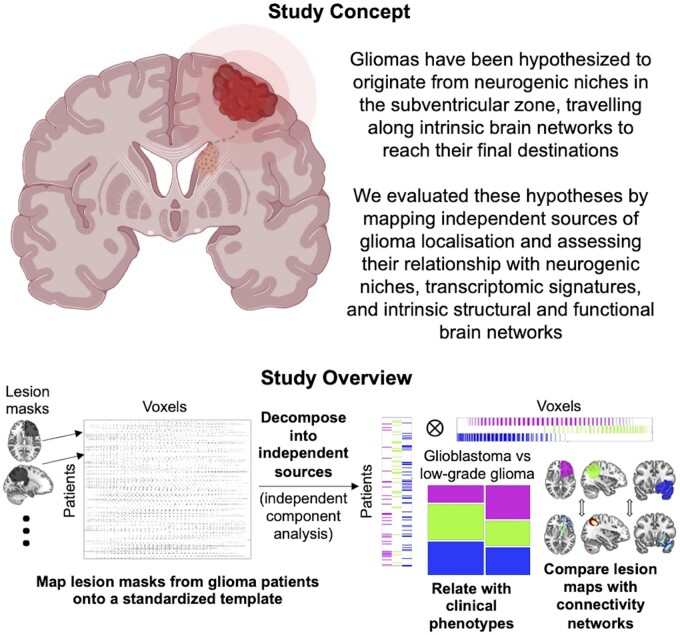

## Introduction

Adult diffuse gliomas are among the most lethal brain disorders, yet the aetiology and pathogenesis of this condition is not well understood. A significant barrier to optimal treatment for gliomas is a lack of clarity regarding the anatomical origins and migration patterns of the tumours. In contrast to early ideas that gliomas originate from mature glial cells in the same locations where they are observed, current theories imply that the tumours originate from neurogenic niches in the subventricular zone (SVZ), from which they then migrate to populate distributed brain areas.^1–3^ This idea is supported by genomic evidence from patients^3^ as well as the observation of significantly elevated glioma frequency surrounding neurogenic niches.^4^^,^^5^ In parallel, other research has indicated that glioma stem cells travel along previously healthy brain structures, including blood vessels and white matter tracts, suggesting that large-scale connectivity networks may help facilitate glioma migration.^6^^,^^7^ However, the pathways by which adult diffuse gliomas could progress from subventricular origins to their final, most commonly cortical,^8^^,^^9^ destinations remain speculative.

The natural progression of other neurological diseases, such as frontotemporal dementias, Alzheimer’s disease and Parkinson’s disease, has been most reliably investigated using longitudinal brain imaging of large cohorts of patients.^10–12^ This approach is difficult to apply to brain tumours, which are typically treated only weeks after initial diagnosis. An alternative method for probing brain development and degeneration from cross-sectional data is the use of structural covariance analysis.^13^ Structural covariance networks identify correlations in brain size (measured by cortical thickness or volume) between brain regions across large cohorts of healthy or diseased individuals.^14^ These interregional relationships reflect a range of shared biological influences, including coordinated development, connectivity and genetic similarity.^15–17^

Analogously, interregional correlations in brain atrophy within defined neurological syndromes have been demonstrated to reflect patterns of coordinated degeneration and network spread of pathology.^18^ Pairs of brain regions which are both consistently affected by a neuropathology could have this relationship for a number of informative reasons, such as a shared biological vulnerability, or a common pathway along which the disease spreads.^19^ The latter possibility is supported by studies of Parkinson’s and Alzheimer’s disease, which have unveiled networks of brain atrophy and tau accumulation that follow intrinsic functional connectivity networks.^20^^,^^21^ The notion that patterns of collateral damage can reveal insights into the aetiology of brain disease is also supported by the phenomenon of lesion covariance in stroke, which stems from the vascular origins of the injury.^22^^,^^23^ An analysis of the networks of brain regions which tend to be co-affected by glioma tumours, therefore, may reveal insights into the possible ventricular origins of these deadly brain cancers, and point to pathways by which the tumours spread throughout the brain.

In this study, we applied independent component analysis (ICA) to tumour masks of patients with low- and high-grade glioma to identify networks of brain regions co-lesioned by gliomas (i.e. lesion covariance networks [LCNs]). Next, we examined associations between these networks and clinically relevant patient information, such as tumour grade, molecular genetics, transcriptomic signature and overall survival. Finally, we related the LCNs to large-scale functional and structural connectivity networks to identify the potential pathways that underlie tumour development. We hypothesized that LCNs would coincide with the three horns of the lateral ventricles, and that the connectivity patterns of periventricular brain regions would correspond with the observed cortical locations of the tumours.

## Materials and methods

**Figure 1:**
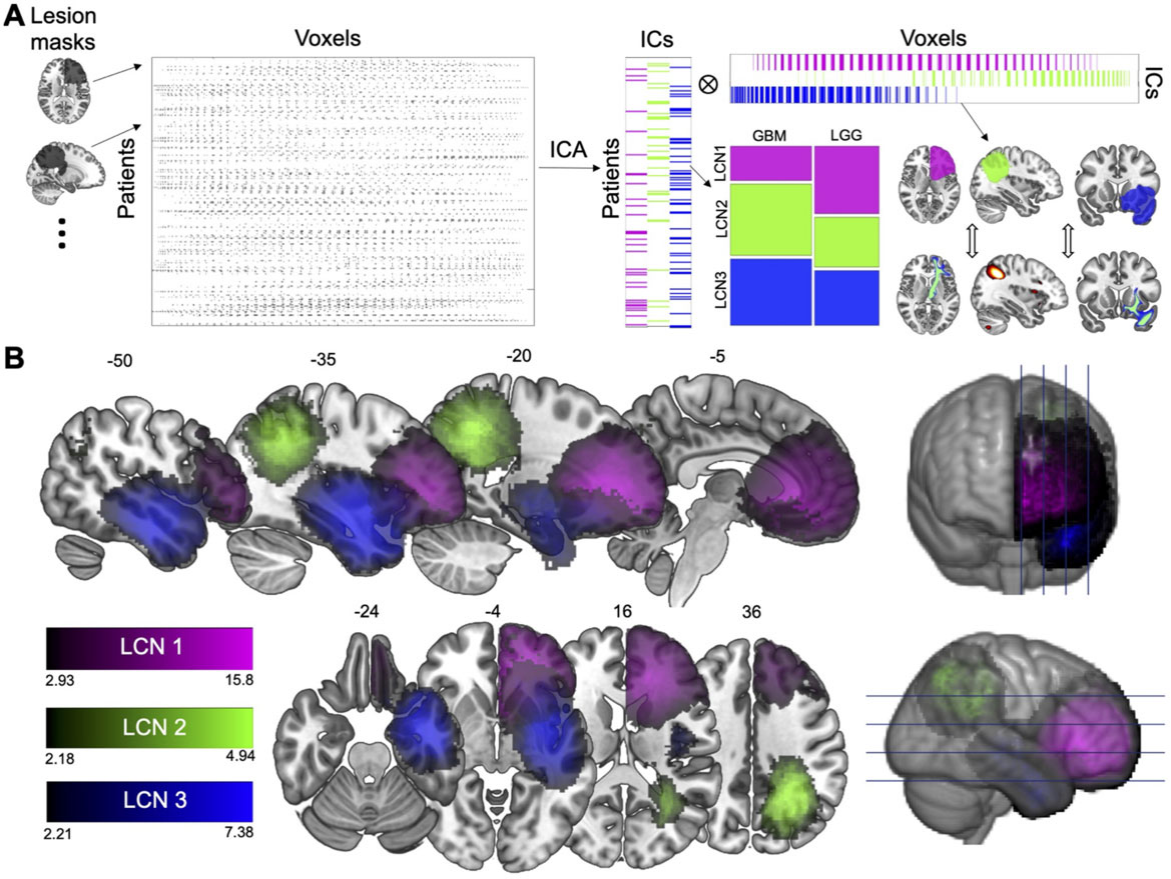
**Lesion covariance networks of glioma localization revealed by ICA.** (**A**) Study workflow. Lesion masks from 242 glioma patients were mapped to one hemisphere then concatenated to form a voxel-wise matrix. This matrix was decomposed via ICA into (i) IC scores, which were related to pathology variables and (ii) spatial maps (i.e. LCNs), which were cross-correlated with structural and functional connectivity networks. (**B**) LCNs are displayed, thresholded to include positive voxels with over 50% likelihood of association with the IC.

A schematic describing the study workflow is provided in Fig. 1A.

### Construction of lesion covariance networks

Three dimensional neuroimaging data of patients with low- and high-grade glioma were accessed from The Cancer Imaging Archive (TCIA; www.cancerimagingarchive.net).^24^^,^^25^ This imaging dataset is linked to The Cancer Genome Atlas (TCGA), which links each scan with genomic, pathological and clinical information regarding the patient. Pre-operative multimodal (i.e. T1w, T1w-Gd, T2w and T2w-FLAIR) scans were obtained from 135 patients with high-grade gliomas and 108 patients with low-grade gliomas (LGG) at 13 institutions with different imaging sequences and protocols (mix of 1.5 T and 3.0 T scans). These scans were skull-stripped, co-registered and resampled to 1 mm^3^ voxel resolution before being entered into GLISTRboost, 26 a top-ranked tumour segmentation algorithm, which classified voxels into four classes: contrast-enhancing tumour, necrotic non-enhancing core, peritumoural oedema and normal brain tissue. Labels were then manually corrected by board-certified neuroradiologists. All preprocessing described above was performed outside the current study and is described in more detail in a prior publication.^25^

Demographic information of the patient sample is included in Table 1. To limit our study to supratentorial lesions, we removed one subject with a posterior fossa tumour. No lesions were multifocal, 38 lesions were ‘butterfly’ gliomas which cross to the opposite hemisphere, and each lesion overlapped with the cortex to some degree. All patients were diagnosed with a diffuse glioma of grade II or higher.

**Table 1:** Demographic, clinical, and imaging variables for 242 patients with glioma from TCIA

Variables of interest	Mean (SD)
Demographic variables	
Age (years)	52.9 (15.2)
Gender (M/F/NA)	133/107/2
Clinical variables	
Grade (GBM/LGG)	135/107
Molecular subtype (IDH-wt/IDH-mut-1p19q-codel/ IDH-mut-1p19q-noncodel/NA)	124/27/61/30
Imaging variables	
LCN groups (LCN1/LCN2/LCN3)	69/87/86
Tumour volume (cm^3^)	52.4 (45.1)

M = male; F = female; NA = not applicable; SD = standard deviation.

Patients were split into groups based on their diagnosis of a glioblastoma (GBM) or LGG, as well as their molecular genetic subtyping (isocitrate dehydrogenase [IDH]-wild-type [wt], IDH-mut/1p19q codeleted and IDH-mut/1p19q non-codeleted). Because this dataset contains patient diagnoses over a long time span (from 1997 to 2013), no consistent diagnostic guidelines were applied for all patients. However, because the 2021 World Health Organization Classification of Central Nervous System Tumour categorizes patients purely by molecular genetics,^27^ the results based on the most recent guidelines can be inferred from the genetic subtyping. Furthermore, we show in Supplementary Fig. 1 that the inclusion of grade III gliomas into the LGG group does not substantially affect the results.

T1-weighted images from each patient were nonlinearly warped to the Montreal Neurological Institute 152 template using Advanced Normalization Tools software, with cost-function masking of abnormal brain tissue.^28^ Accuracy of nonlinear registration was checked manually. Registered masks corresponding to contrast-enhancing tumour and the non-enhancing core were taken to represent, and henceforth will be referred to as, the tumour mask. To reduce the dimensionality of the data, each tumour mask in the right hemisphere was flipped to the left hemisphere, such that each mask was aligned to the same cerebral hemisphere.

Tumour masks from the 242 subjects were combined into one 4D data structure and then entered into MELODIC (Multi-Exploratory Linear Optimized Decomposition of Independent Components) ICA in the FMRIB (Functional Magnetic Resonance Imaging of the Brain) Software Library (FSL).^29^^,^^30^ ICA is a source separation algorithm that decomposes a dataset into a fixed number of statistically independent components (ICs). Given our hypothesis that tumours would stem from the anterior, posterior and inferior horns of the lateral ventricles, we selected a dimensionality of three. The output of ICA included three brain maps of Z statistics indicating the likelihood of each voxel belonging to the corresponding IC, as well as three vectors indicating a score for each patient representing their tumour’s spatial association with each IC. Probabilistic maps for each lesion covariance network were generated using a mixture modelling approach,^31^ and were used to threshold each IC map at 0.5, excluding voxels with a higher likelihood of belonging to a background noise class than to the IC.

### Replication in an independent sample

To determine whether the lesion covariance networks identified in the TCIA dataset could be replicated, we performed ICA on images from the Brain Tumour Segmentation 2019 dataset.^25^^,^^32^^,^^33^ This dataset includes manually segmented images from 335 low- and high-grade glioma patients, pre-processed in the same way as the TCIA dataset described above. We removed patients who overlapped between the two datasets, resulting in 168 subjects. The resulting spatial maps from ICA with three dimensions were cross-correlated with the LCNs from the TCIA dataset, and are displayed in Supplementary Fig. 2.

### Applying LCN mapping to GBM and LGG cohorts separately

We conducted a supplemental analysis to determine whether substantially different results are obtained if the above methodology is applied to GBM and LGG lesions separately. Three LCNs were obtained from the 135 subjects from GBM cohort and 107 subjects from the LGG cohort, respectively, and are displayed in Supplementary Fig. 3.

### Relating LCNs to clinical variables

To determine how the LCNs related to clinically relevant information from the same cohort—including tumour grade, molecular genetics and overall survival—we first assigned each patient to one of the three groups based on the LCN for which their tumour had the highest IC score (i.e. the LCN with which their tumour was most associated). To assess the possible ambiguity of such assignments, we plotted the relationships between LCN values across subjects, finding that no subjects exhibited high values for multiple LCNs simultaneously (Supplementary Fig. 4). Then, we used Chi-square tests to assess the association between these location-based groups and pathology variables such as tumour grade (GBM versus LGG) and molecular genetic subtype (IDH-wt versus IDH-mutant/1p19q codeletion versus IDH-mutant/1p19q non-codeletion). To compare overall survival outcomes between individuals in each LCN group, we plotted a Kaplan–Meier curve. Finally, we performed Cox proportional hazards regressions to quantitatively assess the relationship between LCN group and overall survival. Two models were considered: the first model included LCN group (with LCN3 as the reference level) and demographic covariates (gender and age [binned by the median]); the second model included LCN group, demographic covariates and pathology variables (tumour grade and molecular genetic subtype). Clinical and demographic data were accessed from TCGA.^34–36^ For each analysis, patients with missing data were excluded, resulting in different sample sizes for different tests. These analyses were also performed on the GBM-only and LGG-only cohorts separately (Supplementary Table 1).

### Bulk transcriptomic analyses

To determine if tumours corresponding to different LCNs possessed distinct transcriptomic signatures, we performed bulk RNA-sequencing (RNA-seq) analyses to relate LCN groups to differential gene expression. Following a previously reported workflow,^34^ we downloaded 516 LGG and 155 GBM primary solid tumour samples, 106 and 29 of which could be matched to MRI scans we had for the TCIA-LGG and TCIA-GBM datasets, respectively. To remove potential outliers, we performed an Array–Array intensity correlation, which resulted in a square matrix denoting the Pearson correlation across genes between each TCGA sample (GBM and LGG). No samples were removed after applying a previously established correlation threshold (*r* > 0.6). Next, we normalized our RNA-seq data using the EDAseq package, implementing: (i) within-lane normalization to adjust for the effects of GC-content on read counts; (ii) loess robust local regression, global scaling and full quantile normalization^37^; and (iii) between-lane normalization to adjust for differences between lanes, such as sequencing depth. Finally, we filtered out mRNA transcripts with a quantile mean threshold of 0.25 across all samples, reducing the number of genes considered from 198 66 to 14 899.

Using the edgeR package,^38^ we ran three differential expression analyses comparing between patients included and not included in each LCN group (e.g. LCN1 versus LCN2 and LCN3, etc.). Negative binomial generalized linear models were fit with tagwise dispersion estimated. For each differential expression analysis, genes were ranked by their log_2_ fold-changes, then entered into gene set enrichment analysis (GSEA) to find enriched gene sets associated with each LCN.^39^ Using Cytoscape and Enrichment Map,^40^^,^^41^ GSEA results were displayed as an annotation module network, where enriched gene sets are plotted as nodes and the similarity between gene sets is represented as edges. Because gene sets downregulated for one LCN tended to be upregulated in another, we only plotted positively enriched gene ontologies. These analyses were also performed on the GBM-only and LGG-only cohorts separately.

### Connectivity analyses

We hypothesized that the LCNs would relate to large-scale functional and connectivity networks involved in guiding the development of the tumours. We accessed 21 functional connectivity and 12 structural connectivity networks derived from UK BioBank neuroimaging data of over 4000 neurologically healthy individuals.^42^ Functional connectivity networks were identified from a 25-dimensional ICA decomposition of resting-state fMRI data. Four non-neuronally driven components were excluded. Structural networks were identified using XTract, an automated tractography protocol to identify major white matter pathways with standardized seed, exclusion and termination masks.^43^ We considered four association fibres (inferior fronto-occipital fasciculus, uncinate fasciculus (UNC), inferior and superior longitudinal fasciculus), five projection fibres (acoustic radiation, corticospinal tract, anterior, posterior and superior thalamic radiations) and two limbic fibres (cingulum, main part and hippocampal part). Each of these tracts has left and right counterparts; therefore, streamline density maps from the left and right tracts were aligned to the same hemisphere and averaged. Functional connectivity networks, which also present bilaterally, were similarly mapped to one hemisphere and averaged.

The correspondence between LCNs and structural connectivity networks was quantified by calculating a voxel-wise Spearman’s rank correlation between maps, whereas LCN and functional connectivity correspondence was assessed using Pearson’s correlations. The statistical significance of brain map correspondence was determined by comparing the empirical correlation coefficient with coefficients derived from correlations with 10 000 spatial autocorrelation-preserving surrogate LCN maps generated by BrainSMASH44 (one-sided, non-parametric test). This approach addresses the important confound of spatial autocorrelation to allow for an accurate *P*-value estimation. Specifically, a *P*-value was obtained by comparing the correlation between a given connectivity network and given LCN with the correlations between the same connectivity network and surrogate LCNs with preserved spatial autocorrelation to the original (see Supplementary Fig. 5 for a detailed schematic). A prior study has demonstrated that this approach performs similarly compared with the spin test,^45^ another popular approach for brain map statistical comparison.^46^ For the comparisons between LCNs and functional connectivity, we correlated values corresponding to the cortical and subcortical grey matter voxels of each brain map. Comparisons between LCNs and structural connectivity involved voxels with >1% of the total number of streamlines identified by the XTract protocol.

Finally, we systematically performed seed-based functional connectivity (SBFC) analyses with each subcortical grey matter voxel and correlated the resulting maps with each LCN to identify structures that drive the correspondence between connectivity and lesion covariance. For each voxel in the Harvard–Oxford Subcortical Atlas, we calculated functional connectivity between the subcortical voxel and each cortical grey matter voxel, using the principal components of the UK BioBank Dense Functional Connectome.^47^ The resulting cortical SBFC maps were then normalized using the Fisher *Z*-transformation, and smoothed at 5-mm full-width half maximum. For each LCN, *P*-values were assigned to each subcortical voxel based on the significance of the relationship between its SBFC map and the LCN map using BrainSMASH. The three resulting subcortical *P*-maps were then thresholded to control for multiple comparisons (voxel-wise *P* <0.001; cluster-level family-wise error corrected *P*< 0.05).

### Statistical analysis

All statistical analyses were performed using either MATLAB 2019a or R version 4.0.3. Chi-square tests were used to test for associations between LCNs and pathology variables. Associations between LCNs and overall survival were tested using Cox proportional hazards regression with adjustment for potential confounding variables. Correspondences between structural/functional connectivity networks and LCNs were statistically assessed using one-sided, non-parametric tests involving spatial autocorrelation-preserving surrogate maps,^44^ with false-discovery rate correction for multiple comparisons. Correspondences between LCNs and SBFC maps from subcortical seed voxels were assessed for significance using the same non-parametric approach, controlling for multiple comparisons using permutation-based cluster-level family-wise error correction.^48^

### Data availability

Anonymized lesion data for GBM and LGG, respectively, are available at: https://wiki.cancerimagingarchive.net/display/DOI/Segmentation+Labels+and+Radiomic+Features+for+the+Pre-operative+Scans+of+the+TCGA-GBM+collection and https://wiki.cancerimagingarchive.net/display/DOI/Segmentation+Labels+and+Radiomic+Features+for+the+Pre-operative+Scans+of+the+TCGA-LGG+collection. Clinical and genomic data from TCGA can be downloaded in R by following the workflow described in https://www.bioconductor.org/packages/release/workflows/vignettes/TCGAWorkflow/inst/doc/TCGAWorkflow.html. UK BioBank neuroimaging data are available at: https://www.fmrib.ox.ac.uk/ukbiobank/.

## Results

### Lesion covariance networks implicate horns of the lateral ventricles

We applied ICA to spatially aligned masks of tumour volume derived from a validated imaging processing pipeline applied to pre-surgical brain MRIs of 242 high-grade and LGG patients.^25^ ICA identified three ICs with scores across patients and voxels (Fig. 1A). Given the similarity of the methodological approach to functional connectivity and structural covariance analyses, we decided to refer to the resulting ICs as lesion covariance networks.

ICA revealed three LCNs which extended into the frontal, parietal, and temporal lobes, respectively (Fig. 1B). Notably, each LCN overlapped with a distinct horn of the lateral ventricles, with LCN1 covering the anterior horn, LCN2 covering the posterior horn and LCN3 covering the inferior horn.

**Figure 2:**
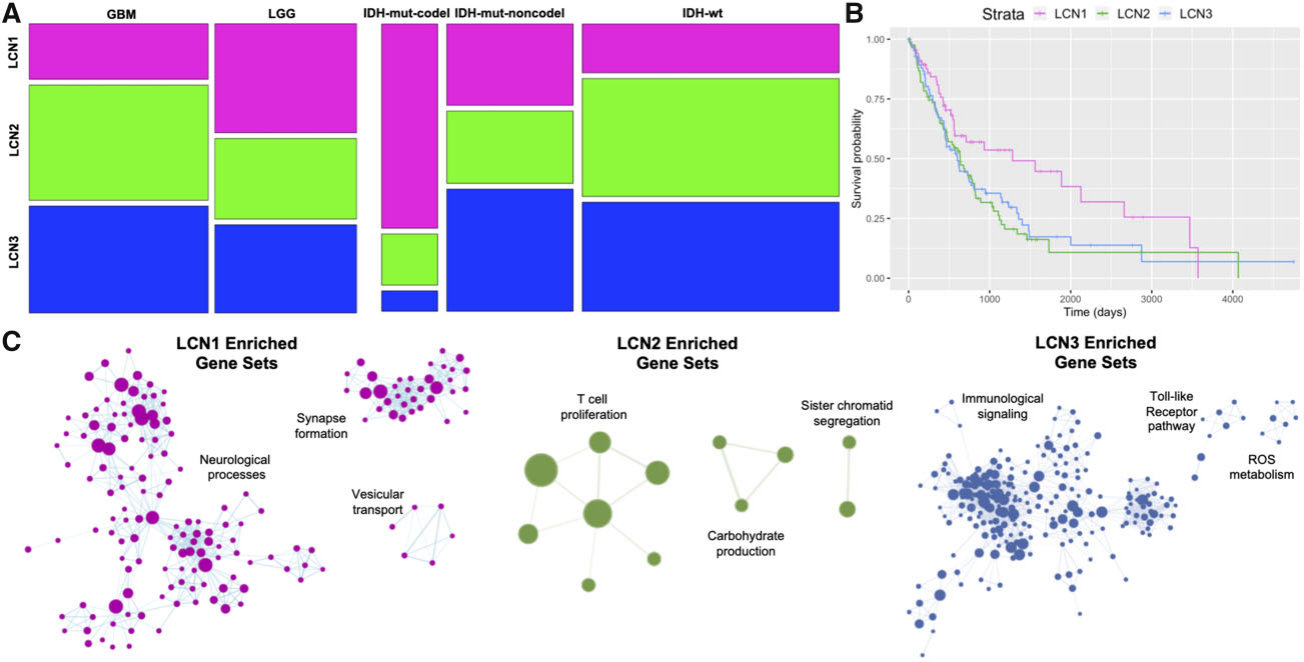
**Clinical and genomic correlates of LCNs.** (**A**) Mosaic plots represent the proportion of gliomas within each LCN associated with pathology variables, including tumour grade and molecular subtype. (**B**) Kaplan–Meier curves show overall survival outcomes stratified by LCN group. (**C**) Gene ontology networks associated with differentially expressed genes for each LCN. Enriched gene sets are plotted as nodes, with gene set size proportional to node size, and the similarities between gene sets are represented as edges. Network components with the three highest numbers of nodes are displayed.

The same LCN locations were replicated in an independent set of 168 glioma patients (*P* < 0.0001 for all matching LCNs; Supplementary Fig. 2). We also observed similar LCN locations, with each component remaining in contact with the lateral ventricles, when the cohort was split into GBM and LGG subsets (Supplementary Fig. 3). Interestingly, a frontal component does not appear when ICA is applied to the GBM-only cohort alone, instead replaced by an LCN contacting the body of the lateral ventricle. This finding is likely the result of reduced representation of LCN1 tumours among GBM lesions (Fig. 2A).

### Clinical outcomes associated with LCNs

To determine how the LCNs related to important pathology variables such as cellular pathology and molecular genetics derived from the same patient sample, we first assigned each patient to one of three groups based on the LCN with which their tumour was most associated. Chi-square tests indicated significant associations between LCN group and tumour grade (χ^2^(2) = 11.1; *P* = 0.0038), as well as between LCN group and IDH/1p19q-status (χ^2^(2) = 6.7; *P* = 0.03) (Fig. 2A). *Post*  *hoc* tests with Bonferroni correction indicated that LCN1 was significantly overrepresented in LGG (Pearson residuals = 3.29; *P* = 0.0059) and IDH-mutated/1p19q-codeleted tumours (residuals = 5.65; *P* < 1e-6), but underrepresented in GBM (residuals = *−*3.29; *P* = 0.0059) and IDH-wt tumours (residuals = *−*4.05; *P* = 0.00046). LCN2 was positively associated with IDH-wt tumours (residuals = 2.84; *P* = 0.04), whereas LCN3 was negatively associated with IDH-mutated/1p19q-codeleted tumours (residuals = *−*3.39; *P* = 0.006). These results implicate LCN1 as a potential radiological signature of IDH-mutated/1p19q-codeleted status, which is pathognomonic of oligodendroglioma.

Next, we related the LCNs to overall survival, first by visualizing Kaplan–Meier curves stratified by LCN group (Fig. 2B). Patients in the LCN1 group had notably prolonged survival compared with patients in the other groups. This association was confirmed statistically and shown to be independent of potential confounding demographic variables through a Cox proportional hazards regression model (Table 2). Interestingly, the effect of LCN1 was no longer significant after pathology variables (i.e. tumour grade and IDH/1p19q-status) were included in the model, suggesting that the association between LCN group and tumour molecular genetics drove the differences in survival outcome. Ability to achieve gross total resection, which is often easier in the frontal lobe, could also contribute to differences in clinical outcome.^49^^,^^50^ Survival outcomes of the LCN groups roughly reflect previously reported survival outcomes stratified by molecular genetic subtyping, wherein IDH-wt, IDH-mut/1p19q non-codeleted and IDH-mut/1p19q non-codeleted tumours rank in the order of lowest to highest in median overall survival.^36^ LCN groups did not relate significantly with survival outcome when the cohort was stratified into GBM-only and LGG-only cases (Supplementary Table 1).

**Table 2:** Cox proportional hazards models relating LCN group and demographic/clinical covariates with overall survival

Demographic covariates only		OS (*n* = 232, deaths = 145)		Demographic and clinical covariates		OS (*n* = 206, deaths = 121)
	HR	SE(HR)	*P*	HR	SE(HR)	*P*
LCN group						
LCN1	**0.62**	**0.23**	**0.036**	0.89	0.25	0.63
LCN2	1.04	0.18	0.82	1.07	0.21	0.75
LCN3	1 (ref)	–	–	1 (ref)	–	–
Demographics						
Age at diagnosis is above median	**3.07**	**0.17**	**1.8e-10**	**1.83**	**0.20**	**0.0028**
Gender, male	1.18	0.18	0.34	0.95	0.19	0.78
Pathology variables						
GBM	–	–	–	1 (ref)	–	–
LGG	–	–	–	0.57	0.31	0.069
IDH-wt	–	–	–	1 (ref)	–	–
IDH-mut/1p19q-codel	–	–	–	**0.22**	**0.54**	**0.0054**
IDH-mut/1p19q-non-codel	–	–	–	**0.29**	**0.37**	**0.00097**

OS = overall survival; HR = hazards ratio; SE = standard error.

Bold values are significant at p < 0.05.

### LCN groups diverge in their expression of neural versus inflammatory genes

In a subset of patients for whom bulk RNA-seq data were available, we performed differential expression analyses to identify genes upregulated among the primary tumours of each LCN group relative to the other groups. GSEAs were performed on the resulting ranked gene lists, and the enriched ontologies were visualized as a network (Fig. 2C). LCN1 was positively enriched with a large number of gene sets associated with neurological processes, such as synaptic signalling and cognition, as well as ontologies involving synapse formation and vesicular transport. The largest network components, comprised gene sets enriched in LCN2 and LCN3, related respectively to T-cell proliferation and immunological signalling. While gene sets overlapped to some degree between LCN2 and LCN3, positively enriched gene sets were mostly distinct (of 297 total gene sets enriched in either LCN2 or LCN3, 10 were enriched in both). The finding of a neural transcriptomic signature for LCN1 is consistent with prior reports of synaptic enrichment among low-grade tumours and oligodendrogliomas,^51^^,^^52^ which each utilized sample sizes larger than implemented here.^51^^,^^52^ To determine whether these enrichments were exclusively driven by LCN1’s association with pathology, we performed a follow-up GSEA for LCN1 where tumour grade and IDH/1p19q status were included as covariates, and found that LCN1 remained enriched for synaptic signalling ontologies (Supplementary Fig. 6). Similar gene ontologies were observed to be enriched when these analyses were performed on the GBM-only and LGG-only cohorts separately (Supplementary Fig. 7).

### LCNs coincide with large-scale connectivity networks

**Figure 3:**
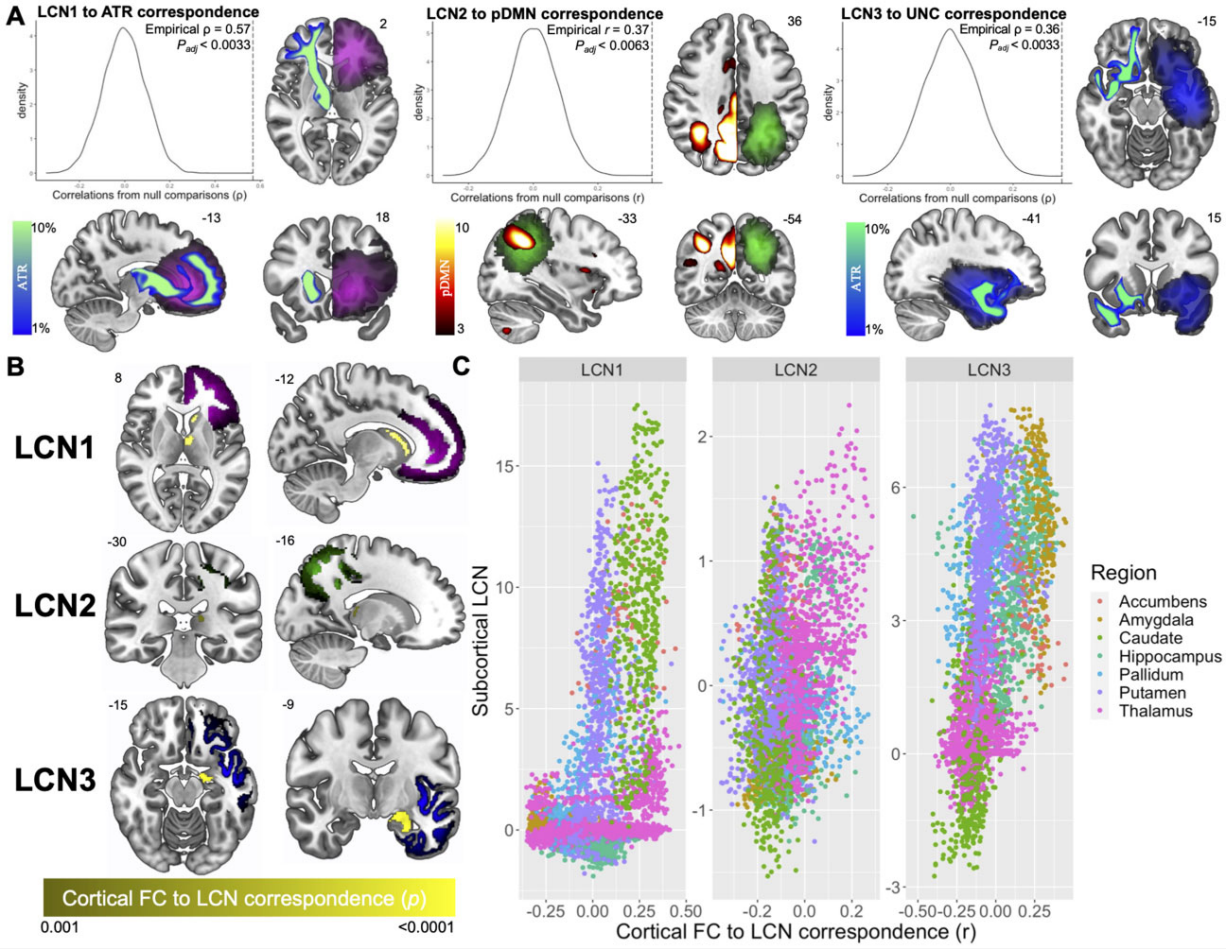
**LCNs of glioma relate to periventricular brain connectivity.** (**A**) Structural and functional connectivity networks with the strongest correspondence with each LCN. Significance of correspondence was assessed by comparison with spatial autocorrelation-preserving surrogate LCN maps.^44^ LCNs are coloured with the same scale as in Figure 1. Structural connectivity networks (where streamline density is represented by a winter colour scale) and functional connectivity networks (where connectivity strength is represented by a hot colour scale) are displayed on the opposite hemisphere of the LCN for visualization in axial and coronal slices. See Supplementary Fig. 8 for other significantly associated connectivity networks. (**B**) Subcortical voxels are coloured based on the significance of the association between their SBFC map and the cortical values of each LCN map (voxel-wise *P* < 0.001; cluster-level *P* < 0.05). The LCNs are also shown with the same colour scale as in Figure 1. (**C**) Scatterplots illustrate subcortical structures with both high functional correspondence and involvement with each LCN, found in the upper right quadrant of each plot.

Next, we assessed anatomical correspondence between LCNs and large-scale connectivity networks, by correlating the three LCNs with 21 functional connectivity networks and 11 white matter pathways derived from a large, healthy neuroimaging dataset.^42^ LCN1 significantly corresponded with four functional connectivity networks each with strong frontal components, including the cingulo-opercular, anterior salience, dorsal attention and frontoparietal networks. LCN1 also corresponded to two major white matter pathways: the anterior thalamic radiation (ATR) and the UNC. LCN2 corresponded with two white matter pathways and two functional networks, including the posterior default mode network (pDMN), whereas LCN3 corresponded with the auditory network. LCNs were mostly related to projection fibres, as well as some longitudinal fibres that connect subcortical and cortical areas, prompting subsequent focus on subcortical–cortical functional connectivity patterns. Effect sizes and *P*-values for all connectivity networks that were statistically significant after correction for multiple comparisons are shown in Table 3. The strongest correspondences between LCNs and large-scale connectivity networks are displayed in Fig. 3A, while other significant associations are shown in Supplementary Fig. 8. In both figures, the connectivity network is displayed on the opposite hemisphere of the LCN purely for visualization purposes—correlations were performed with both networks aligned to the same hemisphere.

**Table 3:** Functional and structural connectivity networks with significant correspondence to LCNs

Functional networks				
LCN	Functional connectivity networks	*R* values	*P* (uncorrected)	*P* (Bonferroni-adjusted)
1	Dorsal attention (IC 7)	0.30	<0.0001	<0.0063
1	Cingulo-opercular (IC 15)	0.44	<0.0001	<0.0063
1	Salience (IC 17)	0.32	<0.0001	<0.0063
1	Fronto-parietal (IC 22)	0.24	0.0003	0.0189
2	Posterior default mode (IC 21)	0.37	<0.0001	<0.0063
3	Auditory (IC 18)	0.28	0.0002	0.0126

Structural networks				

LCN	Structural connectivity networks	*P-*values	*P* (uncorrected)	*P* (Bonferroni-adjusted)

1	Anterior thalamic radiation	0.57	<0.0001	<0.0033
1	Cingulum (main part)	0.09	<0.0001	<0.0033
1	Inferior fronto-occipital	0.31	0.0003	0.0099
1	Uncinate fasciculus	0.49	<0.0001	<0.0033
2	Posterior thalamic radiation	0.32	0.0001	0.0033
3	Acoustic radiation	0.25	0.0001	0.0033
3	Cingulum (hippocampus)	0.21	<0.0001	<0.0033
3	Uncinate fasciculus	0.36	<0.0001	<0.0033

### Cortical LCN locations match functional connectivity with periventricular brain areas

Finally, to identify subcortical structures that may drive the correspondence between connectivity and cortical lesion location, we performed SBFC analyses with all subcortical grey matter voxels and correlated the resulting maps with each LCN. We identified subcortical, periventricular clusters of voxels with cortical functional connectivity patterns that significantly matched each LCN (voxel-wise *P* < 0.001, cluster-level *P* < 0.05; Fig. 3B). To determine the particular structures driving the observed relationships, we generated a scatterplot to highlight subcortical structures with high functional correspondence and involvement with the three LCNs (Fig. 3C), implicating the caudate, thalamus and amygdala, respectively.

## Discussion

In this study, we demonstrated replicable patterns of glioma localization with clinical relevance and spatial correspondence with large-scale functional and structural connectivity networks. Our findings provide evidence for the subventricular origins of glioma, delineate an imaging signature linked to tumour genetics, and contribute to a growing literature on the bidirectional relationship between gliomas and their neural microenvironment.

### Subventricular origins of glioma

Contact with the lateral ventricles is a known prognostic factor for gliomas, predicting poorer overall survival^53–56^ and tumour recurrence.^57^^,^^58^ These observations have motivated the popular notion that neurogenic niches of the SVZ act as a tumour reservoir, contributing to the therapeutic resistance of diffuse gliomas.^59^ However, there remains a point of debate as to whether gliomas spread to the SVZ or if the tumour originates in this area. Our findings inform this debate by establishing: (i) that gliomas cluster around the horns of the lateral ventricles and (ii) that connectivity with periventricular regions corresponds significantly with cortical tumour locations. Our results are most consistent with a model where gliomas originate in any of the three horns of the lateral ventricles, then migrate along neural pathways to arrive at their cortical destinations. The results also suggest that for cases of glioma that appear from radiological imaging to spare the SVZ, the SVZ may nevertheless harbour oncogenic stem cells. This conclusion is consistent with a recent study that found cancer-driving mutations in radiologically tumour-free SVZ tissue in GBM patients.^3^

While our statistical decomposition of glioma distribution captures the lesion locations common to the majority of these tumours, it is worth noting that there are gliomas which do not fit the patterns of the main lesion covariance networks described in this study. For example, none of the LCNs derived from the main cohort substantially covered the supplementary or primary motor areas, where gliomas are sometimes observed. This tumour location did appear in the LCNs of the GBM-only cohort, extending from the central body of the lateral ventricles, but not in the LGG-only or main cohorts. Future work could decompose glioma distribution with a higher dimensionality and subgroup specificity in order to more comprehensively characterize glioma localization patterns.

### Spatial correlates of glioma molecular subtype

The accurate prediction of molecular genetic subtype from tumour imaging is a crucial goal of the burgeoning field of radiogenomics^5^^,^^60–62^ and a potentially transformative clinical tool to aid early and precise glioma diagnosis. Research in this area has illustrated important associations between tumour location and molecular genetic signatures, the most robust of these being a propensity for IDH-mutated gliomas to localize to the rostral end of lateral ventricles.^63^^,^^64^ However, the previous studies in this area used voxel-based lesion symptom mapping, an approach that necessitates stringent multiple comparisons correction,^65^ thereby reducing their power to detect localization differences between subtypes of IDH-mutated tumours. We limited the number of statistical comparisons involved in our study by first reducing the dimensionality of the lesion data. As a result, our method was able to reproduce a previously known association between IDH-mutated/1p19q-codeleted status (pathognomonic of an oligodendroglioma tumour) and lesion location in frontal cortex.^60^^,^^66^ This result is consistent with reports from other, more qualitative neuroimaging studies investigating oligodendroglioma localization.^60^^,^^66^ This replication provides evidence that the presented methodology is suitable for detecting localization differences between lesion subtypes. Similarly, we also note an overlap between LCN3 and the insula, a classic GBM location with notoriously poor outcomes.

Our findings also suggest that tumours with different molecular genetic signatures preferentially arise from different portions of the ventricular lining. A possible explanation for this result is that some gliomas may need a specific metabolic niche in order to thrive and develop into a symptomatic brain tumour. Consistent with this idea, some studies have proposed that high glutamate flux, and the restricted expression of hominoid-specific glutamate dehydrogenase enzymes in the prefrontal cortex, support the survival of IDH-mutant glioma cells in this region.^67^^,^^68^ Thus, the observed localizations of gliomas to particular brain regions or tissue types could provide insight into the aetiology and development of the tumour. Symbiotic relationships between cancer cells and the tumour microenvironment, often framed within the ‘seed and soil hypothesis’, have helped explain the metastatic patterns of other cancers and likely also bear relevance for glioma development.^69^^,^^70^

### Network spread of glioma tumours

Migration of glioma tumours along pre-existing brain structures, including blood vessels and white matter tracts, has been acknowledged since the 1930s.^71^^,^^72^ Over the last decade, it has been demonstrated that migration along these structures is not simply a stochastic process by which tumours follow paths of least resistance.^6^ Rather, glioma cell migration is coordinated, in part, by signalling molecules secreted during neuronal firing, in a process of activity-dependent glial cell proliferation that is also a key mechanism in healthy brain development.^7^^,^^73^^,^^74^ Our findings contribute to this literature by illustrating, in human patients, that glioma localization follows intrinsic functional and structural connectivity networks. This result is consistent with prior work from our group demonstrating that gliomas localize to functional brain hubs.^4^

Early studies of glioma development noted differences between tumour subtypes in their tendency to grow along pre-existing brain structures.^75^ Therefore, some glioma subtypes may be expected to follow brain connectivity networks more closely than others. Our study found support for this idea in that LCN1 related to eight connectivity networks, while the other two LCNs related to just two and four connectivity networks, respectively. Moreover, in addition to possessing the strongest correspondence to brain connectivity, LCN1 was also enriched with genes involved in neuronal processes such as synaptic signalling and synapse formation. A recent study illustrated that glioma cells enriched with these types of genes integrate into neural circuits involved in lexical processing, and that this neural integration supports tumour proliferation.^76^ The genomic signature of a glioma may thus be an important predictor of the tumour’s eventual migration patterns.

We interpret the correspondence between functional connectivity and glioma localization to reflect tumour migration along neuronal networks that support glioma cell proliferation. However, neuronal networks are known to relate intimately with the brain’s vasculature, which has been noted to be a critical spreading substrate for gliomas.^77^ Neuronal and vascular networks converge on similar anatomy in adults,^78^ potentially reflecting the synergistic growth of neuronal and vascular processes during development.^79^^,^^80^ Therefore, one possible interpretation of our results is that the functional networks serve as a proxy for regions with common vascular inputs, and that gliomas invade these territories along the vasculature. This possibility is not mutually exclusive with our primary interpretation, given that neuronal activity could still be driving the migration along blood vessels. The exact physical substrate of activity-dependent glioma cell migration should be investigated further.

## Conclusions

A better understanding of the origins and migration patterns of gliomas could inform surgical and radiation treatments intended to comprehensively obliterate tumour cells. We demonstrated that gliomas cluster around distinct horns of the lateral ventricles, and that these tumour distribution patterns relate to diagnostic genomic signatures and large-scale connectivity networks. Our study connects two separate literatures on the subventricular origins of glioma and symbiotic glioma–neuron relationships to propose a model wherein periventricular brain connectivity guides glioma development.

## Supplementary Material

fcab289_Supplementary_Data

## Abbreviations

GBM =: glioblastoma
ICA =: independent component analysis
IDH =: isocitrate dehydrogenase
LCN =: lesion covariance network
LGG =: low-grade glioma
SVZ =: subventricular zone
