# Supplementary material for: Lesion covariance networks reveal proposed origins and pathways of diffuse gliomas
Source: Brain Commun. 2021 Dec 4;3(4):fcab289. doi: 10.1093/braincomms/fcab289 (PMC8669792; doi:10.1093/braincomms/fcab289)
Supplement: fcab289_Supplementary_Data [file fcab289_supplementary_data.docx]

Lesion covariance networks reveal proposed origins and pathways of diffuse gliomas

Supplementary material

Ayan S. Mandal^1,2^, Rafael Romero-Garcia^1^, Jakob Seidlitz^2,3^, Michael G. Hart^1,4^, Aaron F. Alexander-Bloch^2,3,†^, and John Suckling^1,†^

**^†^These authors contributed equally to this work.**

**Author affiliations:**

1 Department of Psychiatry, Brain Mapping Unit, University of Cambridge, Cambridge, UK CB2 0SZ

2 Department of Psychiatry, Brain-Gene Development Lab, Perelman School of Medicine, University of Pennsylvania, Philadelphia, PA, USA 19104

3 Department of Child and Adolescent Psychiatry and Behavioral Science, Children’s Hospital of Philadelphia, Philadelphia, PA, USA 19104

4 Academic Division of Neurosurgery, Department of Clinical Neurosciences, University of Cambridge, Cambridge, UK CB2 0SZ

Correspondence to: Ayan S. Mandal

Full address: Herchel Smith Building, Robinson Way, Cambridge, UK CB2 0SZ

E-mail: asm82@cam.ac.uk


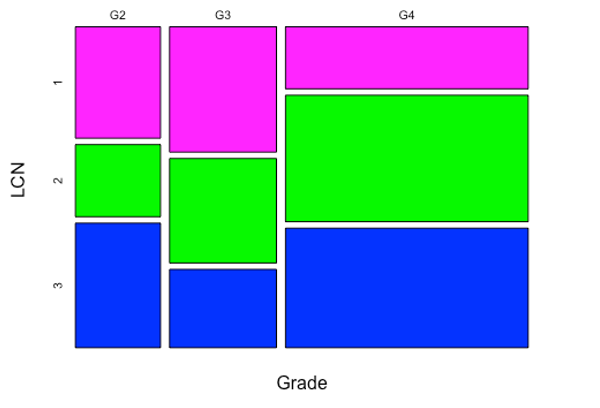


**Supplementary Figure 1. Mosaic plot representing the proportion of patients of each LCN group within each histological grade.**


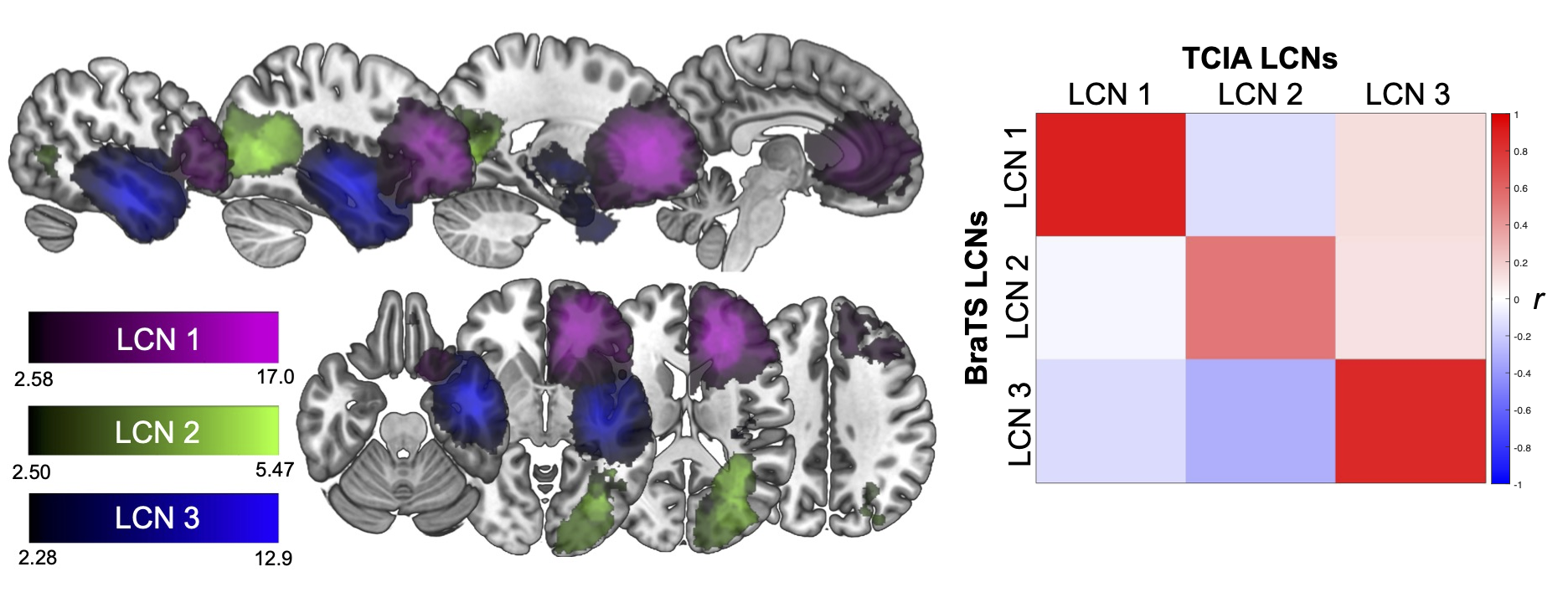


**Supplementary Figure 2. Replication of lesion covariance networks in an independent cohort.** (A) LCNs derived from a cohort of 168 high- and low-grade glioma patients displayed on the same slices shown in Figure 1. BraTS LCNs are coloured identically to their matching LCN in the TCIA cohort in Figure 1. (B) Correlation matrix illustrating the correspondence between LCNs in the TCIA and BraTS cohorts (*P* < 0.0001 for all matching LCNs). Abbreviations: TCIA = The Cancer Imaging Archive; BraTS = Brain Tumor Segmentation Challenge.


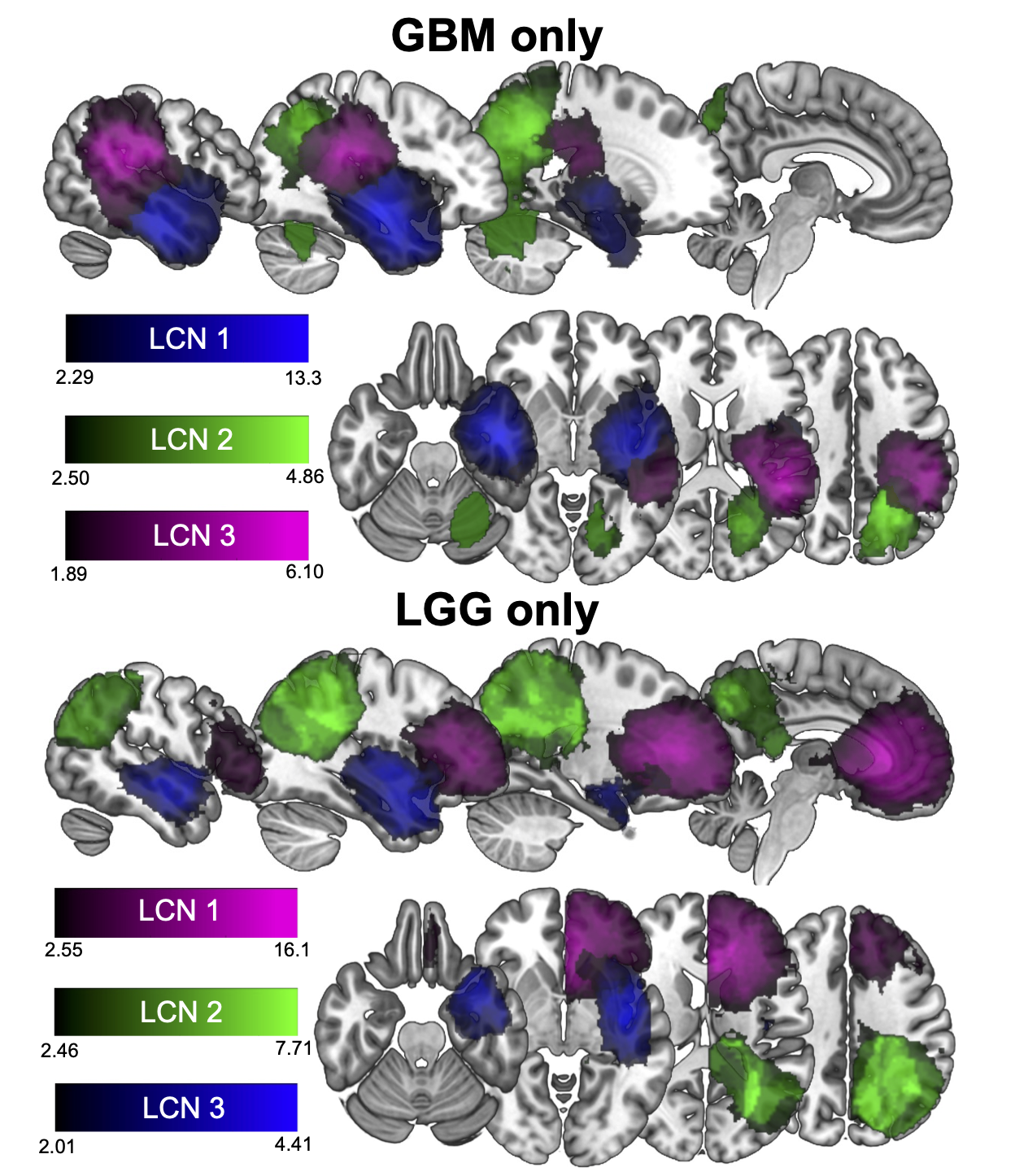


**Supplementary Figure 3. Lesion covariance network mapping of the GBM and LGG cohorts separately.**


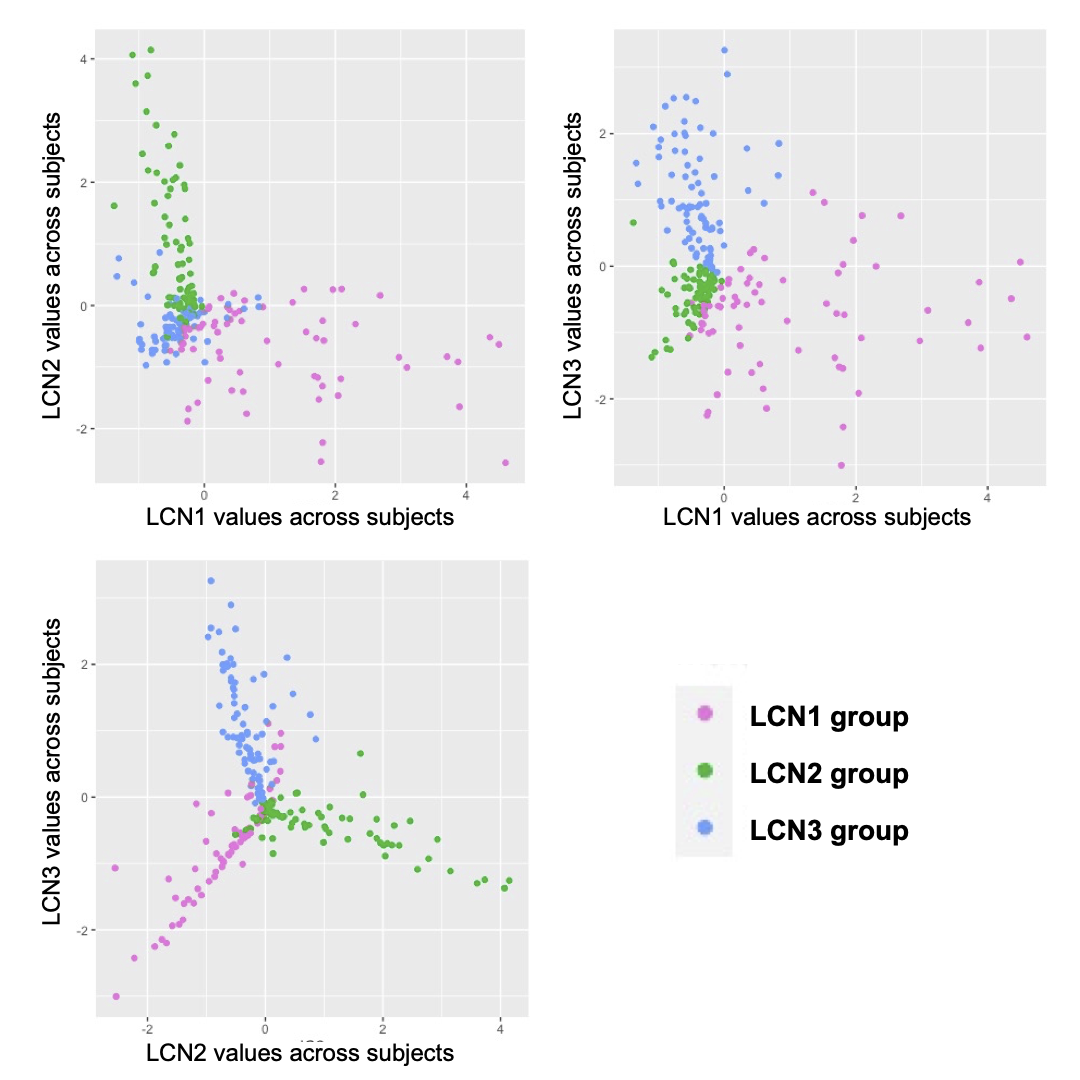
**Supplementary Figure 4. Relationships between LCN values across subjects.**


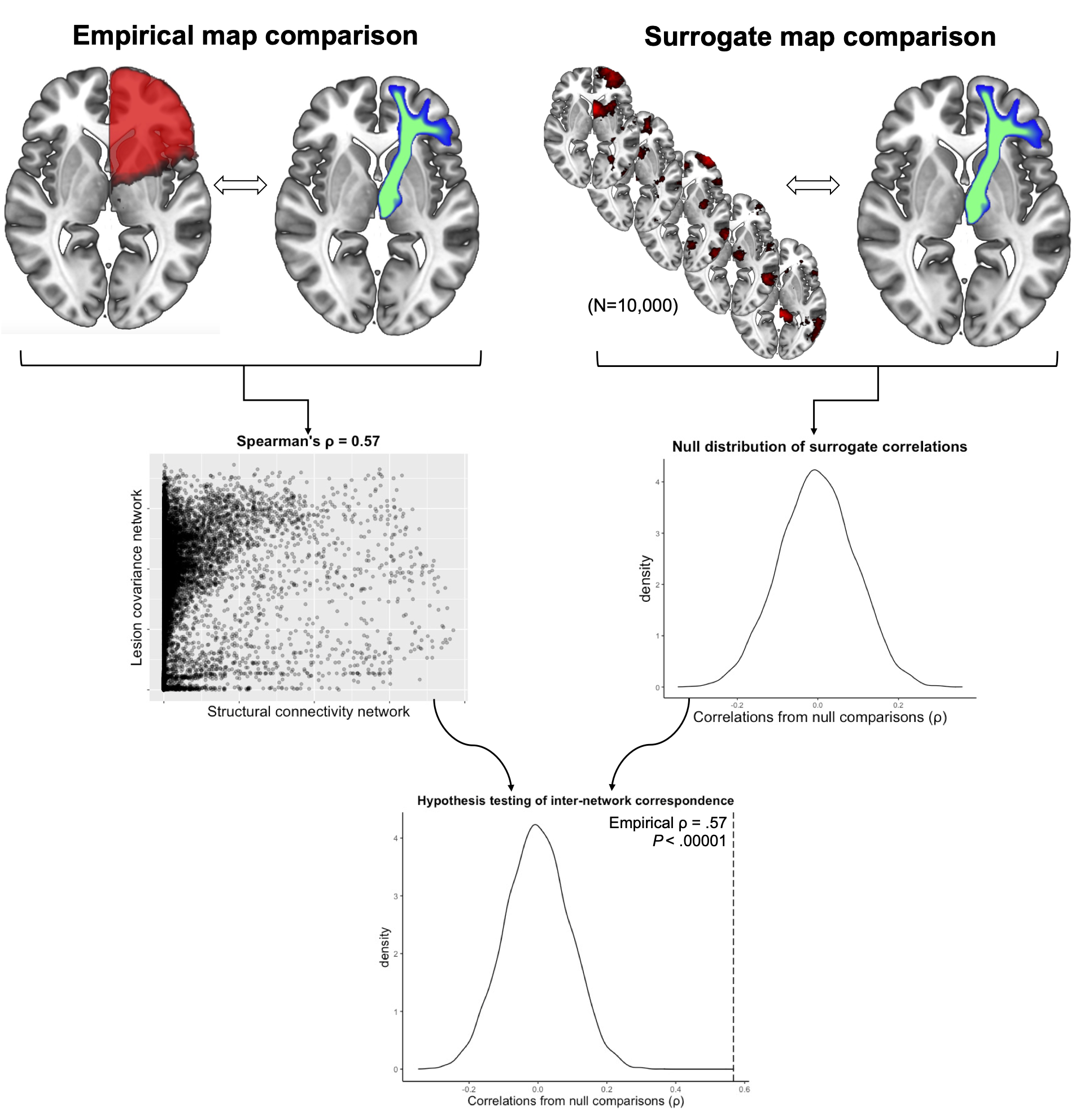


**Supplementary Figure 5. Flow diagram of hypothesis testing for spatial correspondence between brain maps.** First, a correlation is calculated between both maps (top left corner). Second, correlations are calculated between the surrogate brain maps with preserved spatial autocorrelation and the other brain map (top right corner). The null distribution of surrogate correlations is compared with the empirical correlation to determine a *P*-value (bottom).

**
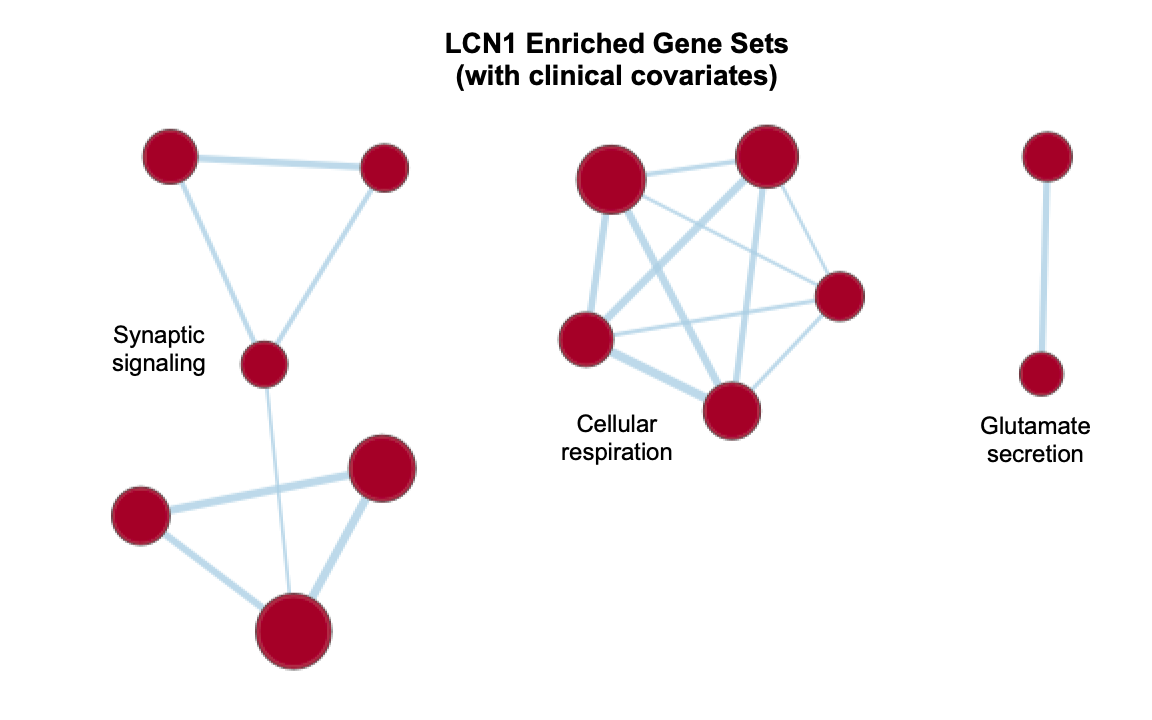
Supplementary Figure 6. Gene ontology networks associated with LCN1 after controlling for tumour grade and molecular genetic subtype.**


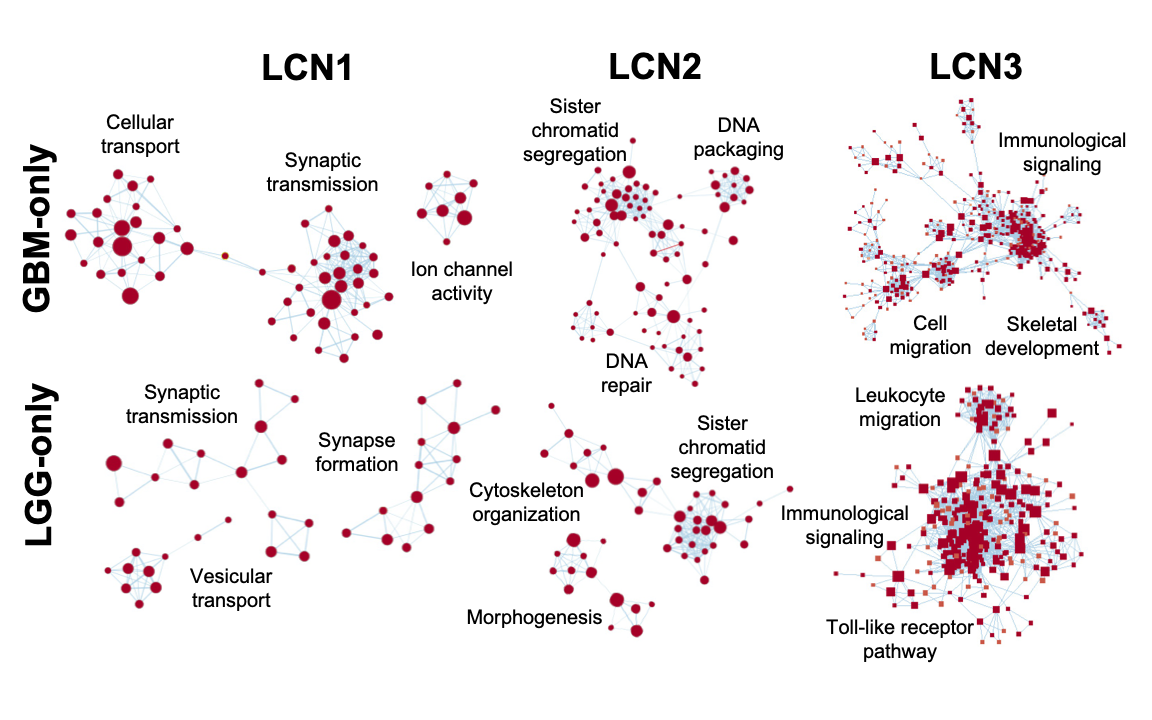
**Supplementary Figure 7. Gene ontology networks associated differentially-expressed genes for each stratified LCN.**

**
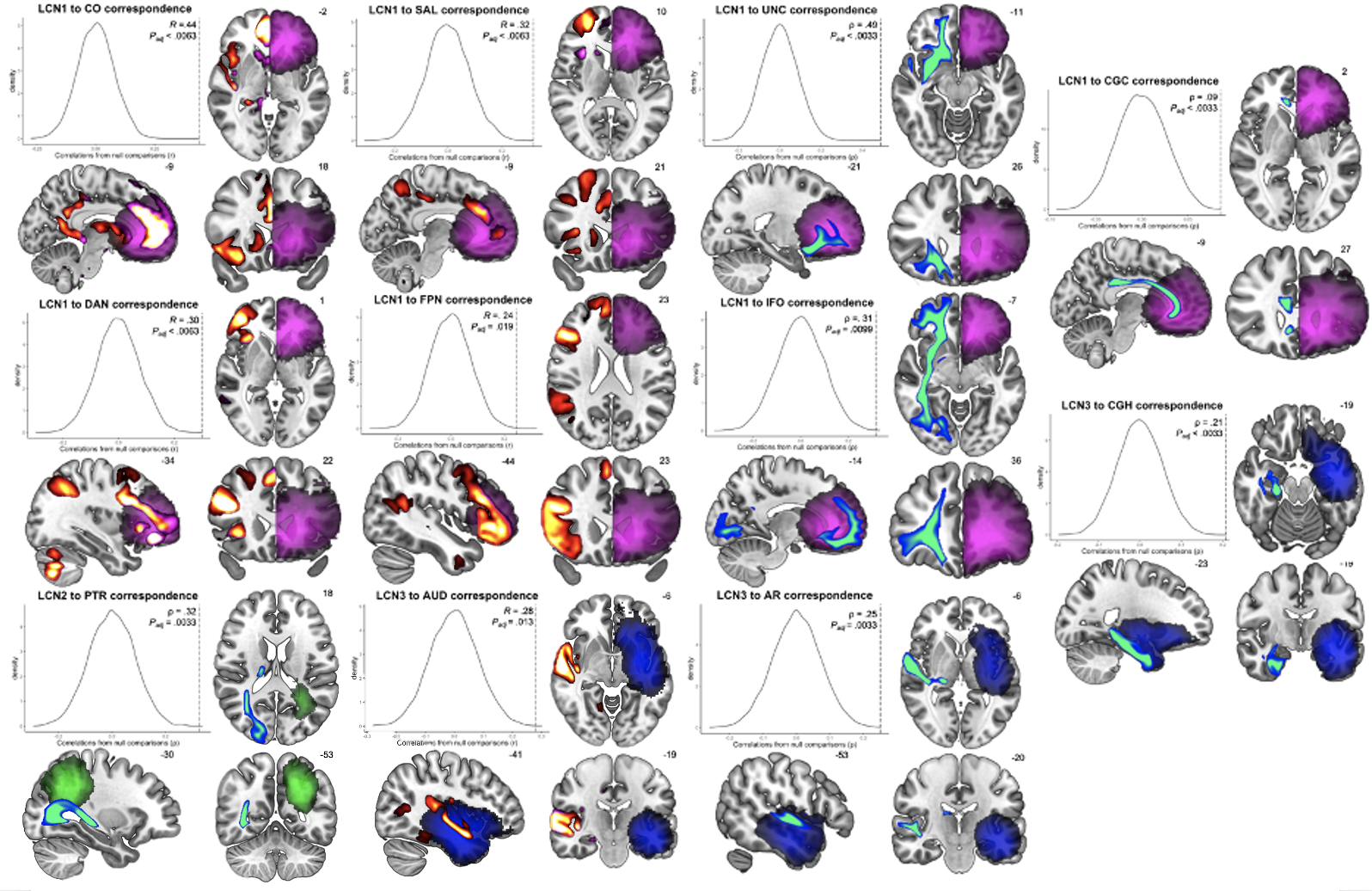
Supplementary Figure 8. Functional and structural connectivity networks significantly associated with lesion covariance networks.**

**Supplementary Table 1. Cox Proportional Hazards models relating stratified LCN groups and demographic/clinical covariates with overall survival.**

| **Demographic covariates only** | | |  | | **Demographic and clinical covariates** | | |  |
| --- | --- | --- | --- | --- | --- | --- | --- | --- |
|  | HR | SE(HR) | | *P* | HR | SE(HR) | *P* | |
| ***GBM-only cohort***  **LCN group** | | | | | | | | |
| LCN1 | 1.27 | 0.23 | | 0.30 | 1.30 | 0.26 | 0.31 | |
| LCN2 | 1.18 | 0.23 | | 0.48 | 1.17 | 0.26 | 0.54 | |
| LCN3 | 1 (ref) | - | | - | 1 (ref) | - | - | |
| **Demographics** |  |  | |  |  |  |  | |
| Age at diagnosis is above median | **1.84** | **0.19** | | **0.001** | **1.58** | **0.21** | **0.029** | |
| Gender is male | 0.86 | 0.20 | | 0.46 | 0.81 | 0.22 | 0.34 | |
| **Pathology variables** | | | | | | | | |
| IDH-wt | - | - | | - | 1 (ref) | - | - | |
| IDH-mut/1p19q-codel | - | - | | - | NA | NA | NA | |
| IDH-mut/1p19q-non-codel | - | - | | - | -1.81 | 0.16 | 0.073 | |
| ***LGG-only cohort***  **LCN group** |  |  | |  |  |  |  | |
| LCN1 | 1.02 | 0.55 | | 0.97 | 1.49 | 0.62 | 0.52 | |
| LCN2 | 1.23 | 0.43 | | 0.62 | 0.58 | 0.51 | 0.28 | |
| LCN3 | 1 (ref) | - | | - | 1 (ref) | - | - | |
| **Demographics** |  |  | |  |  |  |  | |
| Age at diagnosis is above median | 2.00 | 0.38 | | 0.07 | **2.03** | **0.45** | **0.027** | |
| Gender is male | 1.47 | 0.40 | | 0.34 | 2.03 | 0.44 | 0.11 | |
| **Pathology variables** |  |  | |  |  |  |  | |
| IDH-wt | - | - | | - | 1 (ref) | - | - | |
| IDH-mut/1p19q-codel | - | - | | - | **0.10** | **0.68** | **0.00061** | |
| IDH-mut/1p19q-non-codel | - | - | | - | **0.16** | **0.53** | **0.00048** | |

Abbreviations: OS=overall survival; HR=hazards ratio; SE=standard error.
